# Targeting GluN2B/NO Pathway Ameliorates Social Isolation–Induced Exacerbated Attack Behavior in Mice

**DOI:** 10.3389/fphar.2021.700003

**Published:** 2021-07-16

**Authors:** Weiqing Fang, Xiaorong Wang, Miao Cai, Xinxin Liu, Xuemeng Wang, Wen Lu

**Affiliations:** ^1^ Department of Pharmacy, School of Medicine, Women’s Hospital, Zhejiang University, Hangzhou, China; ^2^ Department of Biochemistry and Molecular Biology, School of Basic Medicine and Life Sciences, Hainan Medical University, Haikou, China; ^3^ Department of Clinical Medicine, Hainan Medical University, Haikou, China; ^4^ Key Laboratory of Molecular Biology, School of Basic Medicine and Life Sciences, Hainan Medical University, Haikou, China

**Keywords:** GluN2B, nitric oxide, social isolation, attack, resident–intruder test

## Abstract

Exacerbated attack behavior has a profound socioeconomic impact and devastating social consequences; however, there is no satisfactory clinical management available for an escalated attack behavior. Social isolation (SI) is widespread during this pandemic and may exert detrimental effects on mental health, such as causing heightened attack behavior. To explore the therapeutic approaches that alleviate the SI-induced heightened attack behavior, we utilized pharmacological methods targeting the GluN2B/NO signaling pathway during the attack behavior. Ifenprodil and TAT-9C peptide targeting GluN2B showed that the inhibition of GluN2B mitigated the SI-induced escalated attack behavior and the SI-induced aberrant nitric oxide (NO) level in the brain. Additionally, the potentiation of the NO level by L-arginine reversed the effects of the inhibition of GluN2B. Moreover, we showed that high doses of L-NAME and 7-NI and subeffective doses of L-NAME in combination with ifenprodil or TAT-9C or subeffective doses of 7-NI plus ifenprodil or TAT-9C all decreased the SI-induced escalated attack behavior and reduced the NO level, further supporting the idea that GluN2B/NO signaling is a crucial modulator of the escalated attack behavior.

## Introduction

Attack behavior is an innate behavior that can be useful in obtaining food, protecting territory, and gaining successful mating (Lindenfors and Tullberg, 2011). However, aberrant attacks have a profound socioeconomic impact and devastating social consequences (Liu et al., 2013), yet the clinical management of escalated attack behavior remains unsatisfactory. Stressful life events are commonly accepted as risk factors for the development of psychiatric disorders, including anxiety, depression, and escalated attack behavior (Kessler, 1997; Veenema et al., 2006; Faravelli et al., 2012; Haller et al., 2014). In rodent models, stress paradigms produce a variety of behavioral changes related to clinical symptoms of psychiatric disorders in humans (Hammels et al., 2015; Gururajan et al., 2019). One of the stress paradigms, social isolation (SI) in mice, provides a useful setting in which a stressor can be applied continuously for several days to several weeks (Hawkley et al., 2012). Particularly, SI was widespread in humans in the COVID-19 pandemic and probably had detrimental effects on mental health, and thus possibly elicited exacerbated attack behavior (Banerjee and Rai, 2020; Pietrabissa and Simpson, 2020; Razai et al., 2020; Killgore et al., 2021).

In recent years, the glutamatergic system has been implicated in the development and treatment of emotional and affective disorders (Sanacora et al., 2008; Malgorzata et al., 2020). N-methyl-D-aspartate (NMDA) receptors are the principal glutamate receptors that mediate excitatory synaptic transmission in the mammalian central nervous system (Reiner and Levitz, 2018). NMDA receptors are ionotropic receptors formed by the assembly of four different subunits, consisting of two essential GluN1 subunits and two of the four subunits of the GluN2 subunits (A through D) (Paoletti, 2011; Paoletti et al., 2013). Increasing numbers of the literature demonstrate that NMDA receptors serve a central function in the regulation of emotional and affective behaviors, especially in the attack behavior (Carobrez et al., 2001; Autry et al., 2011; Bortolato et al., 2012). Although numerous results indicate a critical contribution of the NMDA receptor to the attack behavior, the specific role of GluN2B in aggressive behavior has not been fully elucidated. Considering the complex involvement of GluN2B in neuronal functions, it is important to examine the behavioral outcomes of GluN2B blockade in detail and dissect the roles of GluN2B to reveal the clinical relevance and uncover novel therapeutic approaches.

NMDA receptor activation is responsible for nitric oxide (NO) release in the central nervous system (Calabrese et al., 2007). The hyperactivation of the NMDA receptor produces excessive NO, which engenders nitrosative stress in the nervous system, contributing to neurodegenerative damage and affective disorders (Sattler et al., 1999; Girouard et al., 2009; Zorumski et al., 2015). NO, a gaseous free radical that is synthesized by NO synthases (NOS), has been well recognized as a critical neuronal messenger (Bredt and Snyder, 1992; Wink and Mitchell, 1998; Davis et al., 2001). NO affects multiple behaviors by interacting with various proteins in neurons. Studies in rodents have revealed a very complex suite of behavioral changes upon alterations in NO signaling, although the data seem contradictory (Nelson et al., 1995; Demas et al., 1999; Carreno Gutierrez et al., 2020). The complicated and conflicting roles of NO in regulating the attack behavior prompted us to further explore the role of NO in mice.

In the current study, we hypothesized that GluN2B/NO signaling plays a crucial role in mediating the escalated attack behavior induced by SI. To address this, we utilized pharmacological tools to provide evidence that modulation of GluN2B/NO signaling contributes to the escalated attack behavior.

## Materials and Methods

Adult male C57BL/6J mice (weighting 20–25 g) were purchased from SILAC (Shanghai SLAC Laboratory Animal Co. Ltd., Shanghai, China) and housed under controlled conditions (humidity 55–60%; temperature 23 ± 2°C, and 12-h light-dark cycle) with food and water freely available. The number of mice suffering was minimized as possible. All experiments were approved by the Experimental Animal Ethics Committee of Hainan Medical University and the Animal Care and Use Committee of Zhejiang University, which were implemented according to the National Institutes of Health Guide for the Care and Use of Laboratory Animals. The group-housed (GH) mice were 3–4 in a cage, while the social isolation mice were singly housed for 2 weeks.

### Drugs

The drugs were administered through intraperitoneal (IP) or intracerebroventricular (ICV) injection. L-arginine (Sigma, A5006, 20 μg for ICV), L-NAME (Selleck, S2877, 10 μg for effective dose, and 2 μg for subeffective dose by ICV), 7-NI (tsbiochem, T7474, 5 μg for effective dose, 1 μg for subeffective dose by ICV), and ifenprodil (MedChemExpress, HY-12882A, 10 mg/kg for IP, 25 μg for effective dose by ICV, and 5 μg for subeffective dose by ICV) were used. TAT-GluN2B_9C_ peptide (YGRKKRRQRRRKLSSIESDV) or TAT-GluN2B_AA_ peptide (YGRKKRRQRRRKLSSIEADA) was synthesized by Sangon Biotech (Shanghai, China) and injected intraperitoneally (10 mg/kg) or intracerebroventricularly (1 μg for effective dose and 0.2 μg for subeffective dose).

### Guide Cannula Implantation and ICV Injection

The mice were anesthetized with an injection of sodium pentobarbital (50 mg/kg, intraperitoneal injection) and placed in a stereotaxic apparatus. The stereotaxic coordinates for the implantation of the guide cannula into the right lateral ventricle were obtained according to the mouse brain atlas (AP = 0.5 mm relative to the bregma, ML = 0.8 mm relative to the bregma, and DV = −2.5 mm from the skull surface). The guide cannula was then affixed with the dental cement. All injections were carried out over 60 s, and the syringe was left in place for an additional 2 min to minimize backflow after each injection. To verify entry into the right ventricle before our main experiments, 5 μl of trypan blue dye was injected into the cannula, and the mouse brain was cut into slices for observation. Before the initiation of following experiments, animals were allowed 2 weeks to recover after operations. The control animals received the vehicle. During drug infusion, the animals were gently restrained by hand; the stylets were removed from the guide cannula and replaced by 27-gauge injection needles (1 mm below the tip of the guide cannula). Each injection was connected by a polyethylene tube to a 10-μl Hamilton syringe.

### Open Field Test

The open field test was performed as described previously with minor modifications (Ai et al., 2020). Briefly, after 1-h habituation in the testing room, the mice were placed into the corner of a 40 cm × 40 cm × 40 cm square box. The trials were recorded for 10 min. After every trial, the box was cleaned with 75% ethanol to prevent olfactory cues. All data were analyzed using the Super Maze software (Shanghai Xinruan Information Technology Co. Ltd., Shanghai, China).

### Resident–Intruder Test

To assess the aggressive behavior in mice, we used the resident–intruder test. The SI mice were singly housed for 2 weeks, and the GH mice were housed 3–4 mice per cage prior to the introduction of a wild-type male intruder mouse (C57BL/6J, 4 weeks old), which was housed in groups. Behavior was scored blindly for the total number and total duration of the attacks by the resident. An attack was defined as a single bite or a flurry of rapid bites initiated by the resident. The bedding in the cage of the resident mice was not renewed for 3 days before the resident–intruder test.

### Detection of NO

The NO level was measured as described previously with minor modifications (Liu et al., 2015). Immediately after the behavioral test, the cortex of mice was harvested and subjected to the NO detection. The NO level was assayed by measuring the end product nitrite, which was determined based on the Griess reaction. The assay was performed according to the manufacturer’s instructions (S0021S, Beyotime, China). The absorbance of the samples was measured at 540 nm using a microplate reader (BioTek, United States). Nitrite concentration was calculated using a standard curve and expressed as a relative value.

### Statistics

Statistical calculations were performed by GraphPad Prism software (GraphPad Software, Inc. La Jolla, CA). Mean ± SEM was calculated throughout the study, and significance was determined by either two-tailed Student’s t-test or one-way ANOVA with the Bonferroni post hoc test. A *p* value less than 0.05 was considered significant.

## Results

### Blockade of GluN2B-Containing NMDA Receptor by Ifenprodil Mitigates SI-Induced Exacerbated Attack

**FIGURE 1:**
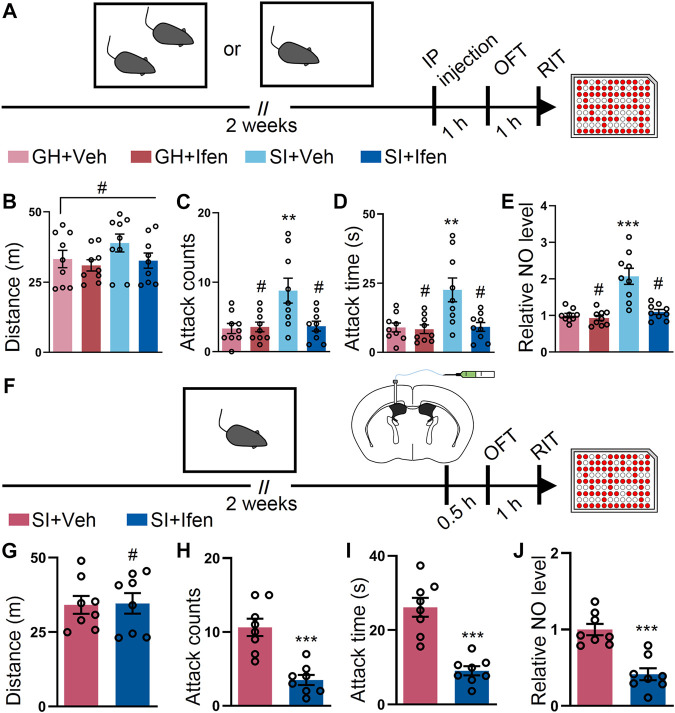
Inhibition of the GluN2B-containing NMDA receptor by ifenprodil mitigates the SI-induced exacerbated attack behavior. **(A)** Schematic of the time course of the experiments. The mice were either group-housed (GH) or single-housed (social isolation, SI) for 2 weeks. After 2 weeks, the mice were intraperitoneally (IP) injected with the vehicle (Veh) or ifenprodil (Ifen) 1 h before the open field test (OFT), and then subjected to the resident–intruder test (RIT) 1 h later. **(B)** Total distance of mice treated as shown in **(A)** during the OFT (F_3, 32_ = 1.536, *p* = 0.224). **(C)** Attack counts of mice treated as shown in **(A)** during the RIT (F_3, 32_ = 5.877, *p* = 0.0026). **(D)** Total attack time of mice treated as shown in **(A)** during the RIT (F_3, 32_ = 7.282, *p* = 0.007). **(E)** The relative NO level of the cortex from mice treated as shown in **(A)** (F_3, 32_ = 18.95, *p* < 0.001). **(F)** Schematic illustrating the experimental procedure. The mice were implanted with a guide cannula and singly housed for 2 weeks. Afterward, ifenprodil was intracerebroventricularly (ICV) injected, and behavioral tests were performed. **(G)** Total distance of mice treated as shown in **(F)** during the OFT (t_14_ = 0.1058, *p* = 0.9172). **(H)** Attack counts of mice treated as shown in **(F)** during the RIT (t_14_ = 5.231, *p* < 0.001). **(I)** Total attack time of mice treated as shown in **(F)** during the RIT (t_14_ = 6.067, *p* < 0.001). **(J)** The relative NO level of the cortex from mice treated as shown in **(F)** (t_14_ = 5.428, *p* < 0.001). Data are represented as mean ± SEM. *p < 0.05, **p < 0.01, ***p < 0.001, and # indicates not significant.

Accumulating evidence indicates that the NMDA receptor plays vital roles in the attack behavior. Therefore, we first examined whether the systematic application of ifenprodil, an inhibitor of GluN2B (Schoemaker et al., 1990), alleviated SI-induced exacerbated attack (Figure 1A). No alterations in the open field were observed, suggesting that ifenprodil had a minimal impact on the locomotor activity of the mice (Figure 1B). As expected, the systematic application of ifenprodil markedly reduced attack counts and duration in the attack assay after SI, while the GH mice with ifenprodil show a similar behavior in numbers and duration in the attack behavior compared to the GH mice with vehicle treatment (Figures 1C,D). We also tested the NO level in the cortex of the GH mice and SI mice with or without ifenprodil treatment. Ifenprodil but not the vehicle treatment elicited a decrement in the NO level in the SI mice (Figure 1E). Additionally, no significant difference in the NO level was detected in the GH mice with or without ifenprodil treatment (Figure 1E). To confirm the central action of the drug, ifenprodil was intracerebroventricularly injected into the SI mice (Figure 1F). As expected, no differences were detected in the open field test after ICV injection (Figure 1G). In line with the results obtained following the systemic administration, ICV injections of ifenprodil provoked a marked reduction in attack behaviors, as shown in attack counts and duration (Figures 1H,I), thus demonstrating that ifenprodil-dependent behavioral effects are brain-mediated. Moreover, the reduced level of NO was restored in the SI mice with an ICV injection of ifenprodil (Figure 1J). Collectively, these results suggest that the blockade of GluN2B ameliorate escalated attack behavior and the aberrant NO level induced by SI.

### Blockade of the GluN2B-Containing NMDA Receptor by TAT-9C Peptide Ameliorates SI-Induced Escalated Attack Behavior

**FIGURE 2:**
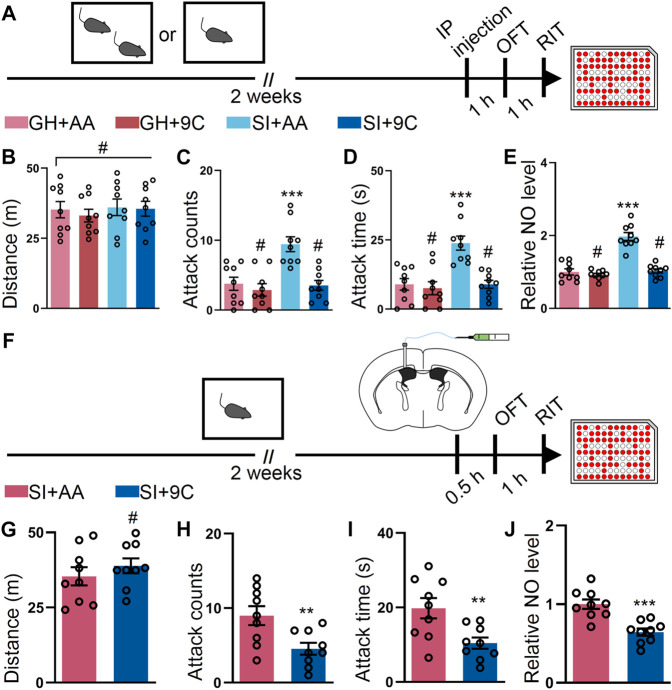
TAT-9C peptide targeting GluN2B-containing NMDA receptor ameliorates the SI-induced escalated attack behavior. **(A)** Time course of the experiments. **(B)** Total distance of mice treated as shown in **(A)** during the OFT (F_3, 32_ = 0.2299, *p* = 0.8748). **(C)** Attack counts of mice treated as shown in **(A)** during the RIT (F_3, 32_ = 11.16, *p* < 0.001). **(D)** Attack duration of mice treated as shown in **(A)** during the RIT (F_3, 32_ = 12.85, *p* < 0.001). **(E)** The relative NO level of the cortex from mice treated as shown in **(A)** (F_3, 32_ = 41.40, *p* < 0.001). **(F)** Schematic illustrating the experimental procedure. The mice were implanted with a guide cannula and singly housed for 2 weeks. Afterward, TAT peptide was injected *via* the ICV route, and behavioral tests were performed. **(G)** Total distance of mice treated as shown in **(F)** during the OFT (t_16_ = 0.9012, *p* = 0.3808). **(H)** Attack counts of mice treated as shown in **(F)** during the RIT (t_16_ = 2.961, *p* = 0.0092). **(I)** Attack duration of mice treated as shown in **(F)** during the RIT (t_16_ = 3.005, *p* = 0.0084). **(J)** The relative NO level of the cortex from mice treated as shown in **(F)** (t_16_ = 4.704, *p* < 0.001). Data are represented as mean ± SEM. *p < 0.05, **p < 0.01, ***p < 0.001, and # indicates not significant. AA represents TAT-AA peptide, while 9C indicates TAT-9C peptide.

To corroborate our results on the GluN2B antagonist, we tested whether downregulation of GluN2B by TAT-9C peptide (Aarts et al., 2002; Zhang et al., 2020) reduced the escalated attack behavior induced by SI. To perform this, we employed the TAT-9C peptide or TAT-AA peptide (Figure 2A). No differences in OFT were detected between the TAT-9C and TAT-AA peptide–treated mice (Figure 2B). As anticipated, the TAT-9C peptide–treated SI mice show reduced attack behavior compared with the TAT-AA peptide–treated SI mice and the GH mice with either TAT-AA or TAT-9C peptide treatment (Figures 2C,D). Accordingly, the NO level was decreased in the TAT-9C peptide–treated SI mice compared with that in the TAT-AA peptide–treated SI mice (Figure 2E). Moreover, ICV injection of the two peptides exhibited similar results as the systematic injection of the two peptides on OFT, RIT, and NO levels (Figures 2F–J). Taken together, these findings provided convergent evidence which supports the notion that GluN2B plays a critical role in the SI-induced heightened attack behavior.

### L-NAME or 7-NI Attenuates the SI-Induced Escalated Attack Behavior

**FIGURE 3:**
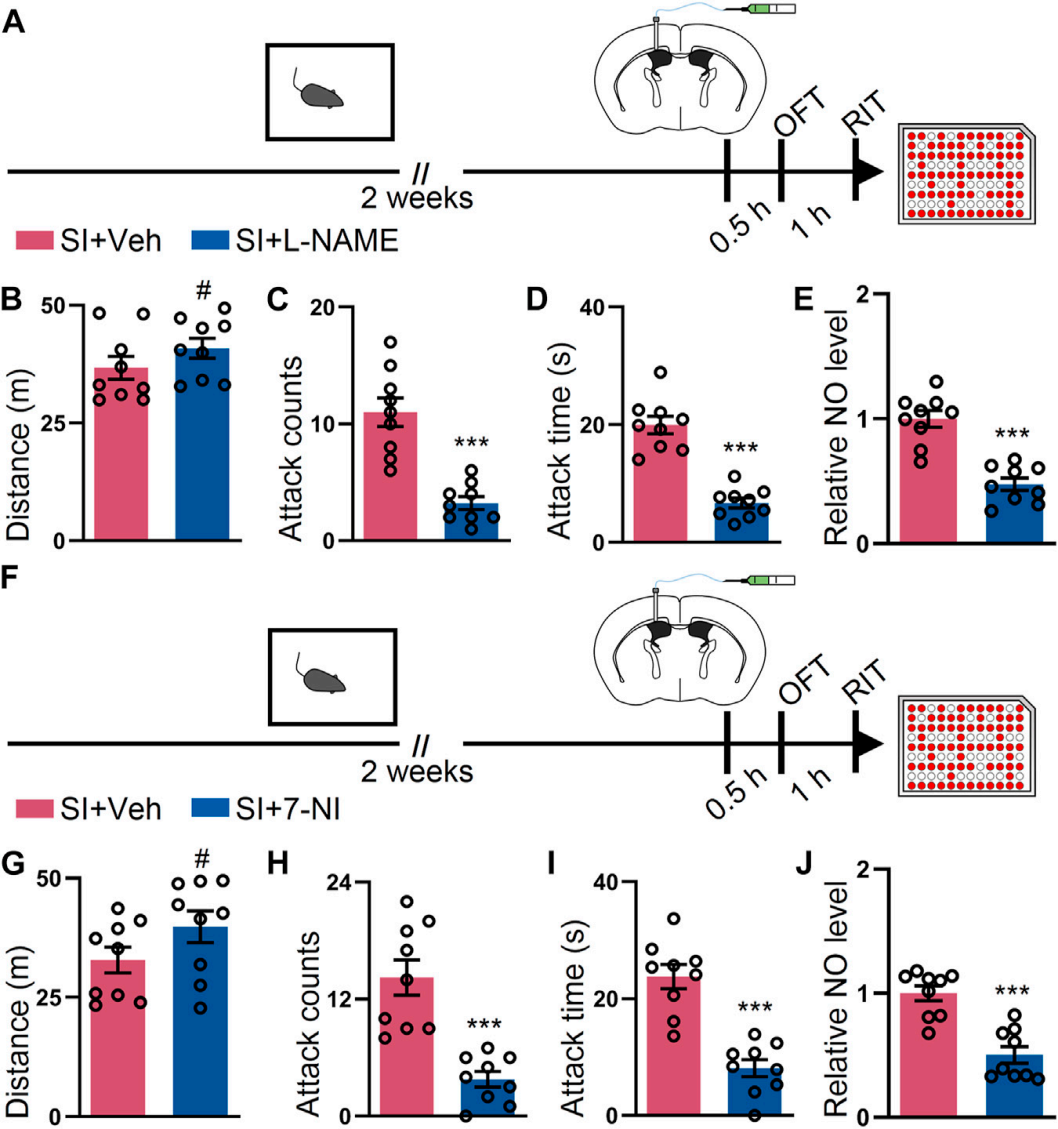
L-NAME or 7-NI blocked SI-induced heightened attack behavior. **(A)** An outline of the experimental procedure for behavioral tests and NO detection. The mice were implanted with a guide cannula and singly housed for 2 weeks. Afterward, L-NAME was injected *via* the ICV route, and behavioral tests were performed. **(B)** Total distance of mice treated as shown in **(A)** during the OFT (t_16_ = 1.279, *p* = 0.2193). **(C)** Attack counts of mice treated as shown in **(A)** during the RIT (t_16_ = 5.798, *p* < 0.001). **(D)** Total attack time of mice treated as shown in **(A)** during the RIT (t_16_ = 7.798, *p* < 0.001). **(E)** The relative NO level of the cortex from mice treated as shown in **(A)** (t_16_ = 6.309, *p* < 0.001). **(F)** An outline of the experimental procedure for behavioral tests and NO detection. The mice were implanted with a guide cannula and singly housed for 2 weeks. Afterward, 7-NI was injected *via* the ICV route, and behavioral tests were performed. **(G)** Total distance of mice treated as shown in **(F)** during the OFT (t_16_ = 1.629, *p* = 0.1229). **(H)** Attack counts of mice treated as shown in **(F)** during the RIT (t_16_ = 5.267, *p* < 0.001). **(I)** Total attack time of the mice treated as shown in **(F)** during the RIT (t_16_ = 6.187, *p* < 0.001). **(J)** The relative NO level of the cortex from mice treated as shown in **(F)** (t_16_ = 5.489, *p* < 0.001). Data are represented as mean ± SEM. *p < 0.05, **p < 0.01, ***p < 0.001, and # indicates not significant.

Given that our data suggest that NO participates in SI-induced elevated attack, we sought out to determine whether inhibition of the NO synthetase (NOS) activity by L-NAME (Pfeiffer et al., 1996) could attenuate the SI-induced escalated attack behavior. To perform this intervention, we intracerebroventricularly injected L-NAME and subsequently conducted the behavioral assay to evaluate the attack behavior (Figure 3A). The inhibition of NOS activity with L-NAME prevented the SI-induced escalated attack behavior but failed to alter the locomotor activity of the mice (Figures 3B–D). Notably, the NO levels in the two groups were monitored by harvesting the cortex after the behavioral assay. L-NAME treatment markedly reduced the level of NO in SI mice (Figure 3E). To dissect the isoform of NOS responsible for the SI-induced attack behavior, we injected 7-NI, a nNOS inhibitor (Moore et al., 1993), by the ICV route and performed the behavioral test (Figure 3F). 7-NI efficiently blocked the SI-induced escalated attack behavior but minimally affected the total distance traveled by the mice during the open field test (Figures 3G–I). Additionally, 7-NI reversed the increment in the NO level induced by SI (Figure 3J). Collectively, our data suggest that the inhibition of NOS activity, particularly the nNOS activity, attenuates the SI-induced escalated attack behavior and deregulated the NO level.

### L-Arginine Reverses the Behavior Effects Exerted by the Inhibition of GluN2B in the SI Mice

**FIGURE 4:**
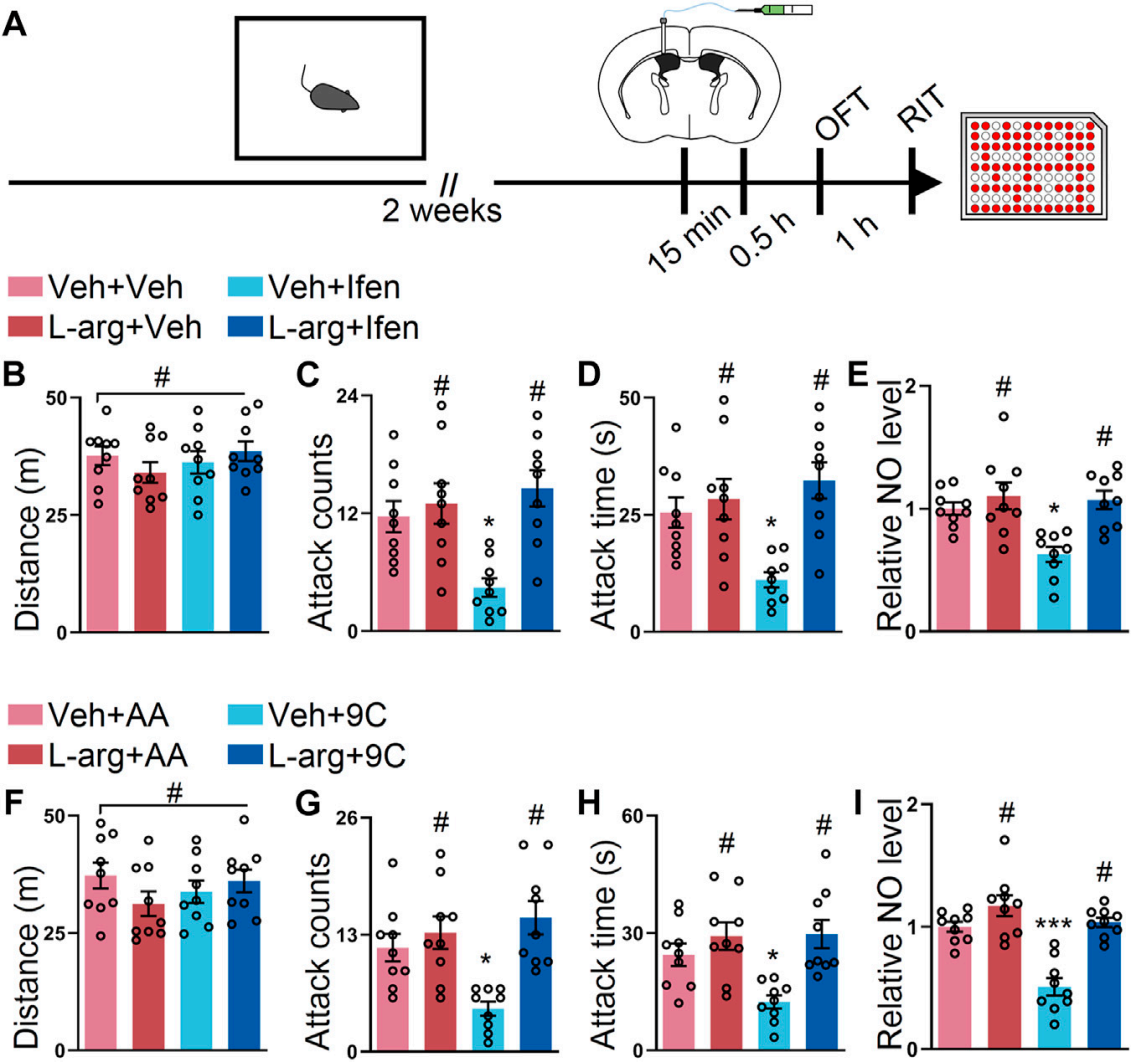
L-arginine reversed the effect provided by blockade of GluN2B on the SI-induced exacerbated attack behavior. **(A)** Schematic diagram of the experimental procedure for behavioral tests and NO detection. The mice were implanted with a guide cannula and singly housed for 2 weeks. Afterward, L-arginine (L-arg) and ifenprodil or L-arginine and TAT peptide were injected *via* the ICV route, and behavioral tests were performed. **(B)** Total distance of the mice that received L-arginine, ifenprodil, or both during the OFT (F_3, 32_ = 0.8199, *p* = 0.4925). **(C)** Attack counts of mice that received L-arginine, ifenprodil, or both during the RIT (F_3, 32_ = 7.254, *p* < 0.001). **(D)** Attack duration of mice that received L-arginine, ifenprodil, or both during the RIT (F_3, 32_ = 7.359, *p* < 0.001). **(E)** The relative NO level of the cortex from the mice that received L-arginine, ifenprodil, or both (F_3, 32_ = 8.158, *p* < 0.001). **(F)** Total distance of the mice that received L-arginine, TAT peptide, or both during the OFT (F_3, 32_ = 1.096, *p* = 0.3651). **(G)** Attack counts of the mice that received L-arginine, TAT peptide, or both during the RIT (F_3, 32_ = 8.164, *p* < 0.001). **(H)** Attack duration of the mice that received L-arginine, TAT peptide, or both during the RIT (F_3, 32_ = 7.150, *p* < 0.001). **(I)** The relative NO level of the cortex from the mice that received L-arginine, TAT peptide, or both (F_3, 32_ = 21.34, *p* < 0.001). Data are represented as mean ± SEM. *p < 0.05, **p < 0.01, ***p < 0.001, and # indicates not significant.

L-arginine is an amino acid catalyzed by NOS to produce NO (Wu and Morris, 1998). To explore the causal relationship between GluN2B/NO signaling and the attack behavior, we injected L-arginine into the SI mice before ifenprodil or the TAT peptide treatment *via* the ICV route (Figure 4A). During the OFT, the mice with either treatment displayed similar locomotor activities (Figure 4B). However, the preinjection of L-arginine abolished the behavioral effects on the mice with ifenprodil injections (Figures 4C,D); however, L-arginine failed to affect the attack behavior of the mice with vehicle injections (Figures 4C,D), possibly due to the ceiling effect of NO on the attack behavior. Besides, the NO level only decreased in the ifenprodil-treated mice (Figure 4E). As shown in Figure 4F, no overt differences were detected in the mice treated with L-arginine and TAT peptide. In the attack behavior, L-arginine prevented the effect of the TAT-9C peptide (Figures 4G,H). Accordingly, L-arginine also reversed the decline in the NO level elicited by TAT-9C treatment (Figure 4I). Together, these data further suggest that GluN2B/NO signaling is involved in the attack behavior.

### Co-Application of L-NAME and Ifenprodil or L-NAME and TAT-9C Provides Additive Effects on the SI-Induced Escalated Attack Behavior

**FIGURE 5:**
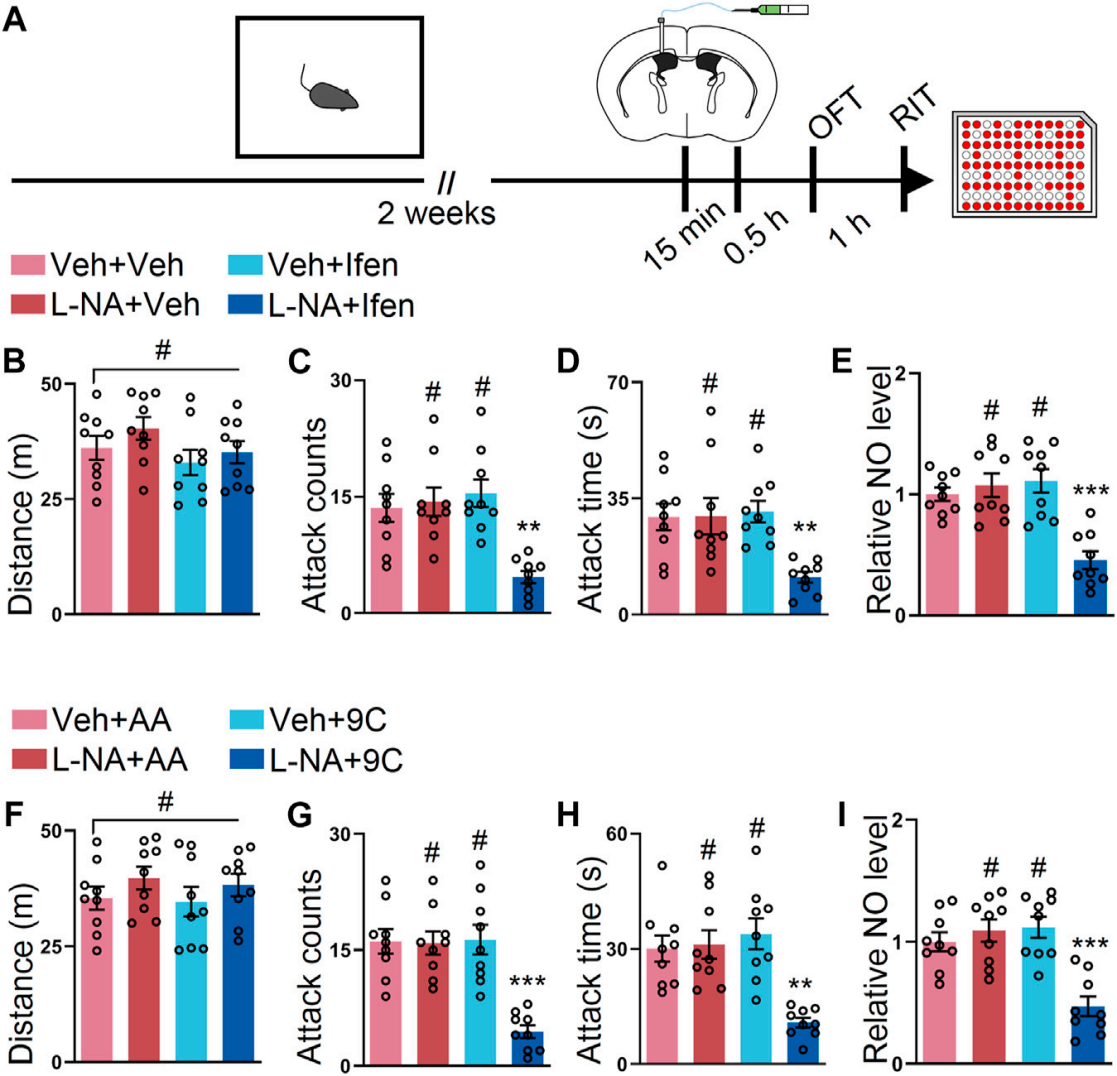
Low dose of L-NAME in combination with ifenprodil or a low dose of L-NAME in combination with TAT-9C corporately reduced the SI-induced heightened attack behavior. **(A)** Schematic drawing of the experimental procedure for behavioral tests and NO detection. The mice were implanted with a guide cannula and singly housed for 2 weeks. Afterward, L-NAME (L-NA) and ifenprodil or L-NAME and TAT peptide were injected *via* the ICV route, and behavioral tests were performed. **(B)** Total distance of the mice that received L-NAME, ifenprodil, or both during the OFT (F_3, 32_ = 1.479, *p* = 0.2388). **(C)** Attack counts of the mice that received L-NAME, ifenprodil, or both during the RIT (F_3, 32_ = 9.454, *p* < 0.001). **(D)** Total attack time of the mice that received L-NAME, ifenprodil, or both during the RIT (F_3, 32_ = 5.988, *p* = 0.0023). **(E)** The relative NO level of the cortex from the mice that received L-NAME, ifenprodil, or both (F_3, 32_ = 13.69, *p* < 0.001). **(F)** Total distance of the mice that received L-NAME, TAT peptide, or both during the OFT (F_3, 32_ = 0.8068, *p* = 0.4994). **(G)** Attack counts of the mice that received L-NAME, TAT peptide, or both during the RIT (F_3, 32_ = 14.91, *p* < 0.001). **(H)** Total attack time of the mice that received L-NAME, TAT peptide, or both during the RIT (F_3, 32_ = 10.24, *p* < 0.001). **(I)** The relative NO level of the cortex from the mice that received L-NAME, TAT peptide, or both (F_3, 32_ = 13.12, *p* < 0.001). Data are represented as mean ± SEM. *p < 0.05, **p < 0.01, ***p < 0.001, and # indicates not significant.

Given that our data demonstrated that separated targeting GluN2B or NOS reduced SI-induced escalated attack, we determined to check whether the simultaneous inhibition of GluN2B and NOS provides additive effects. To test this, L-NAME, ifenprodil, or both were injected into the lateral ventricle at the subeffective dose (Figure 5A). There were no alterations during the OFT in the mice treated with subeffective doses of L-NAME, ifenprodil, or both drugs (Figure 5B). Interestingly, only the co-application of L-NAME and ifenprodil reduced the SI-induced escalated attack behavior, while L-NAME + vehicle or vehicle + ifenprodil administration failed to reduce the SI-induced escalated attack behavior (Figures 5C,D). Indeed, the NO level was only reduced by the co-application of L-NAME and ifenprodil (Figure 5E). Furthermore, we also examined the effects of the co-injection of L-NAME and TAT-9C. The mice with either treatment displayed similar locomotor activities during the OFT (Figure 5F). However, neither L-NAME + TAT-AA nor vehicle + TAT-9C attenuated the SI-induced escalated attack behavior, but the coadministration of L-NAME and TAT-9C efficiently mitigated the SI-induced escalated attack behavior (Figures 5G,H). As expected, the NO level was markedly decreased in the mice with the coadministration of L-NAME and TAT-9C, whereas the NO levels were unaltered in the L-NAME + TAT-AA or vehicle + TAT-9C–treated mice compared with the vehicle + vehicle-treated mice (Figure 5I).

### Co-Application of 7-NI and Ifenprodil or 7-NI and TAT-9C Convey Additive Effects on the SI-Induced Heightened Attack Behavior

**FIGURE 6:**
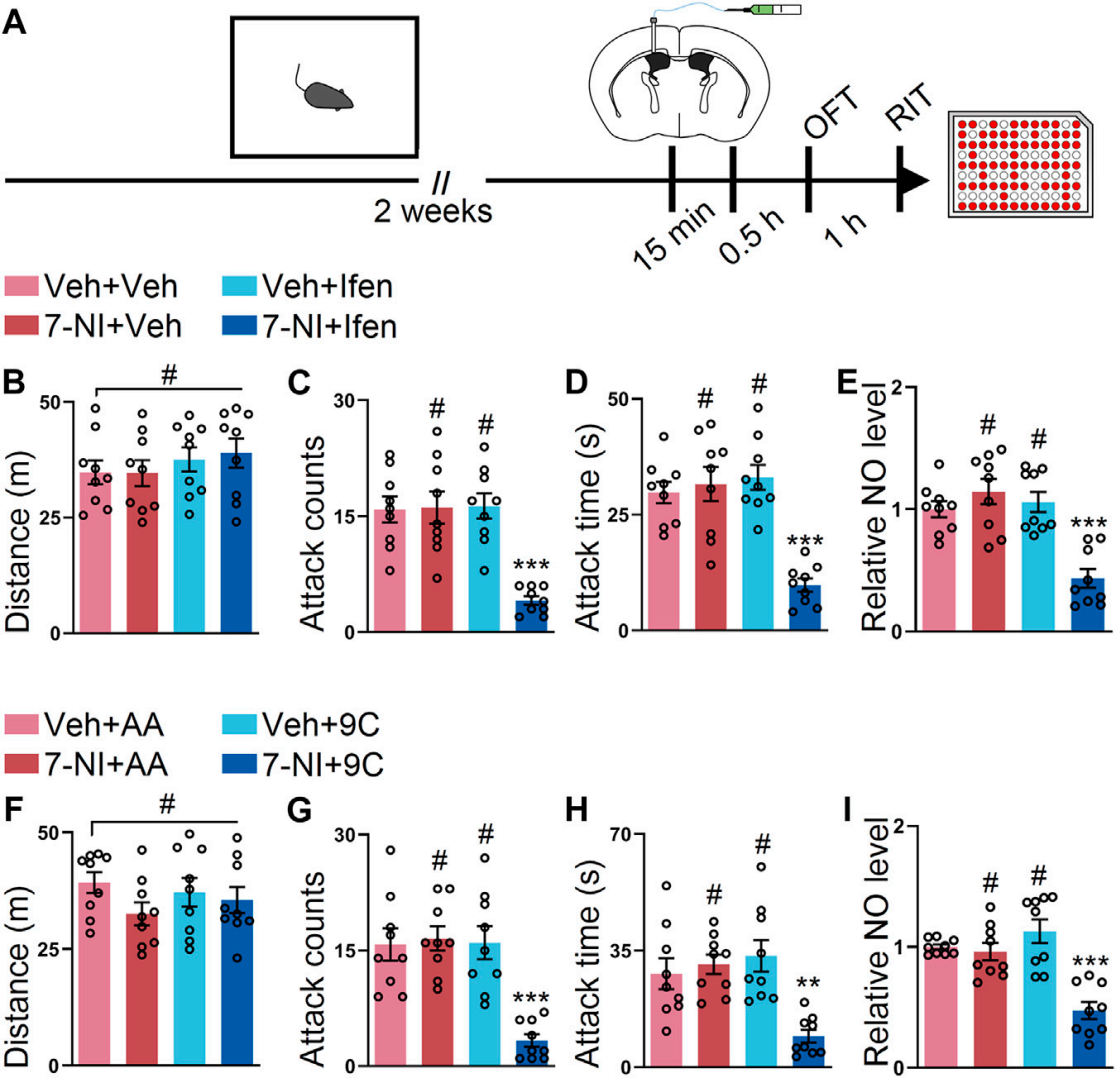
Low dose of 7-NI plus ifenprodil or low dose of 7-NI plus TAT-9C had additive effects on reducing the SI-induced heightened attack behavior. **(A)** An outline of the experimental procedure for behavioral tests and NO detection. The mice were implanted with a guide cannula and singly housed for 2 weeks. Afterward, 7-NI and ifenprodil or 7-NI and TAT peptide were injected *via* the ICV route, and behavioral tests were performed. **(B)** Total distance of the mice that received 7-NI, ifenprodil, or both during the OFT (F_3, 32_ = 0.5849, *p* = 0.6293). **(C)** Attack counts of the mice that received 7-NI, ifenprodil, or both during the RIT (F_3, 32_ = 14.16, *p* < 0.001). **(D)** Attack duration of the mice that received 7-NI, ifenprodil, or both during the RIT (F_3, 32_ = 16.82, *p* < 0.001). **(E)** The relative NO level of the cortex from the mice that received 7-NI, ifenprodil, or both (F_3, 32_ = 14.97, *p* < 0.001). **(F)** Total distance of the mice that received 7-NI, TAT peptide, or both during the OFT (F_3, 32_ = 1.133, *p* = 0.3503). **(G)** Attack counts of the mice that received 7-NI, TAT peptide, or both during the RIT (F_3, 32_ = 13.52, *p* < 0.001). **(H)** Attack duration of the mice that received 7-NI, TAT peptide, or both during the RIT (F_3, 32_ = 8.508, *p* < 0.001). **(I)** The relative NO level of the cortex from the mice that received 7-NI, TAT peptide, or both (F_3, 32_ = 16.27, *p* < 0.001). Data are represented as mean ± SEM. *p < 0.05, **p < 0.01, ***p < 0.001, and # indicates not significant.

Finally, we sought out to determine whether 7-NI and ifenprodil or 7-NI and TAT-9C also had an additive effect on the SI-induced escalated attack behavior. To address this issue, 7-NI, ifenprodil, or both drugs were injected into the lateral ventricle at the subeffective dose (Figure 6A). The results from the OFT showed that the mice with the vehicle, subeffective doses of L-NAME, ifenprodil, or both drugs had comparable distances (Figure 6B). Intriguingly, only the co-application of 7-NI and ifenprodil could downregulate the SI-induced heightened attack behavior, while the subeffective doses of 7-NI or ifenprodil administration failed to reduce the SI-induced escalated attack behavior (Figures 6C,D). As shown in Figure 6E, the NO level was only reduced by the co-application of 7-NI and ifenprodil. Furthermore, we examined the effects of the co-injection of 7-NI and TAT-9C. The mice displayed comparable locomotor activities during the OFT with either treatment (Figure 6F). As expected, the subeffective doses of 7-NI or TAT-9C had a minimal impact on the SI-induced heightened attack behavior, but the coadministration of 7-NI and TAT-9C at subeffective doses efficiently blocked the SI-induced escalated attack behavior (Figures 6G,H). Accordingly, the NO level was significantly decreased in the mice treated with coadministration of 7-NI and TAT-9C, whereas the NO level was unchanged in the 7-NI + TAT-AA or vehicle + TAT-9C–treated mice compared to the vehicle + TAT-AA–treated mice (Figure 6I). Together, these data suggest that GluN2B/NO signaling plays a key role in the SI-induced heightened attack behavior, and the coadministration of the inhibitors of GluN2B and NOS at subeffective doses could block the SI-induced escalated attack behavior.

## Discussion

In the present study, we provided convergent evidence that GluN2B/NO signaling participated in the SI-induced escalated attack behavior. Pharmacological tools using the GluN2B-specific antagonist ifenprodil and TAT peptide indicated that GluN2B played a critical role in the SI-induced exacerbated attack behavior and SI-elicited aberrant NO levels. Additionally, the potentiation of the NO level by L-arginine reversed the effects provided by the inhibition of GluN2B on the SI-induced heightened attack. Moreover, L-NAME and 7-NI decreased the SI-induced escalated attack behavior, further supporting the idea that NO is a crucial modulator of the escalated attack behavior. Our data are in consistent with those of previous reports showing GluN2B and NO are involved in the attack behavior. In addition, the drugs (ifenprodil, TAT peptide, L-NAME, and 7-NI) used in the experiments impact minimal effect on the locomotor activities of the mice, which excludes the influence of the locomotor differences in the attack behaviors observed.

NMDA receptors, a major type of ionotropic glutamate receptor, have long been recognized to be involved in neuropsychiatric disorders (Lakhan et al., 2013). Previous studies have revealed that NMDA receptors in the prefrontal cortex, amygdala, and ventral hippocampus are engaged in the attack behavior (Bortolato et al., 2012; Chang et al., 2015; Bacq et al., 2020; Nordman et al., 2020). Additionally, the escalated attack behavior in rats with peripubertal stress is associated with an increment of the mRNA encoding an obligatory subunit of NMDA receptor, GluN1, in the amygdala (Tzanoulinou et al., 2014). Therefore, accumulating evidence demonstrates that NMDA receptors are profoundly involved in the escalated attack behavior (Bortolato et al., 2012; Chang et al., 2018). The data in this study support this notion and further indicate that the GluN2B subunit of the NMDA receptor gates the SI-induced elevated attack behavior. Although other potential signaling molecules within the GluN2B/NO pathway remain to be uncovered, one of the major pathways downstream of NMDA/nNOS is a carboxyl-terminal PDZ ligand of neuronal nitric oxide synthase protein (CAPON)/dexamethasone-induced ras protein 1 (Dexras1), which is an alternative promising therapeutic target for treating mood disorders, such as anxiety (Zhu et al., 2014; Zhu et al., 2020). According to recent reports, Zlc002, a fast-acting prodrug without sedative and myorelaxant effects, blocks the nNOS/CAPON interaction and has significant anxiolytic-like effects in rodents (Zhu et al., 2014; Zhu et al., 2020). Thus, it is interesting to explore the effects of Zlc002 on the SI-induced heightened attack behavior in the future. Nevertheless, our results show that the GluN2B/NO pathway is indispensable in the SI-induced escalated attack behavior in mice. Considering that GluN2B is vital in SI-induced escalated attack, targeting GluN2B for alleviation of the escalated attack behavior is reasonable and promising. For this purpose, several pharmacological agents, such as TAT-9C peptide, serve as potential therapeutic drugs. The TAT-9C peptide, which disrupts the association between GluN2B and PSD95, was reported to be safe and efficient for ischemic stroke in a phase-2 clinical trial (Hill et al., 2012; Wu and Tymianski, 2018). To our knowledge, our study is the first to reveal the potential of TAT-9C as a therapeutic drug to treat the escalated attack behavior, and thus, it is worthy to test in clinical trials in the future.

The hyperfunction of NMDA receptors results in an abnormal NO level in the cortex in several neurological diseases (Zhou et al., 2010; Ghasemi and Dehpour, 2011; Qu et al., 2020). NO, as a second messenger, which is synthesized in response to neuronal activation, acts as an important modulator of neuronal function that is involved in synaptic plasticity, depression, and aggressive behavior (Shibuki and Okada, 1991; Nelson et al., 2006; Steinert et al., 2010; Luscher and Malenka, 2012). The roles of NO in the attack behavior are conflicting, possibly due to the housing conditions of the animals used in the studies, the genetic background of the animals, source of NO, or unpredicted side effects caused by genetic manipulation (Carobrez et al., 2001; Autry et al., 2011; Bortolato et al., 2012; Gao and Heldt, 2015). Nevertheless, our data suggest that nNOS plays a predominant role in the heightened attack behavior in the SI mice. However, the involvement of other NOS isoform, eNOS or iNOS, in the attack behavior remains to be determined in the future. Several lines of evidence indicate that the cortex, including its various subregions, is involved in the attack behaviors (De Almeida et al., 2006; Cambon et al., 2010; Chiavegatto et al., 2010; Biro et al., 2018; van Heukelum et al., 2021). Therefore, we measured the NO levels in the cortex in the present study. However, other brain regions, such as the amygdala or bed nucleus of the stria terminalis (BNST), also participate in the attack behavior (Wang et al., 2013; Nordman et al., 2020). Hence, further studies are warranted to provide a comprehensive view of the NO in different regions of the brain in the future.

The injection route used in the current study was IP or ICV. Therefore, the specific brain region in which the drug acted remains to be uncovered. Generally, we demonstrated that the systematic application of the drugs can ameliorate the SI-induced escalated attack behavior. Moreover, to circumvent the adverse side effects elicited by high doses of the GluN2B antagonist or NOS antagonist, the co-application of the GluN2B antagonist and NOS antagonist at subeffective doses may be an alternative for treating the escalated attack behavior.

## Data Availability

The original contributions presented in the study are included in the article/Supplementary Material; further inquiries can be directed to the corresponding authors.
